# Small Circular Rep-Encoding Single-Stranded DNA Genomes in Peruvian Diarrhea Virome

**DOI:** 10.1128/genomeA.00822-17

**Published:** 2017-09-21

**Authors:** Eda Altan, Juana Del Valle Mendoza, Xutao Deng, Tung G. Phan, Mohammadreza Sadeghi, Eric L. Delwart

**Affiliations:** a Blood Systems Research Institute, San Francisco, California, USA; b Department of Laboratory Medicine, University of California at San Francisco, San Francisco, California, USA; c School of Medicine, Research and Innovation Centre of the Faculty of Health Sciences, Universidad Peruana de Ciencias Aplicadas, Lima, Peru; d Instituto de Investigación Nutricional, Lima, Peru; e Department of Virology, University of Helsinki, Helsinki, Finland

## Abstract

Metagenomic analysis of diarrhea samples revealed the presence of numerous human enteric viruses and small circular Rep-encoding single-stranded DNA (CRESS-DNA) genomes. One such genome was related to smacoviruses, while eight others were related to genomes reported in the feces of different mammals. The tropism of these CRESS-DNA viruses remains unknown.

## GENOME ANNOUNCEMENT

Fecal samples from 300 Peruvian patients with diarrhea were pooled in groups of 10 and processed to enrich for viral particle-associated nucleic acids using filtration and nuclease treatment. Amplification of nucleic acids using random reverse transcription (RT)-PCR was followed by use of the Illumina Nextera method to generate sequencing libraries, which were sequenced using the MiSeq instrument (250-bp paired-end reads) (1). *De novo* assembly was performed using the Ensemble program (2). The resulting singlets and contigs were analyzed using BLASTx to search for similarity to viral proteins in GenBank’s Virus RefSeq.

Numerous human viruses were identified with E scores of <10^−30^, including anelloviruses, adenovirus D, *Caliciviridae* (Sapporo virus, Norwalk virus), *Picornaviridae* (enterovirus A/B/C, parechovirus, aichivirus A, cosavirus C/D/E, salivirus, cardiovirus), *Astroviridae* (mamastrovirus 1, astrovirus MLB1), *Parvoviridae* (bocavirus 1, bufavirus 3, adeno-associated virus), and picobirnavirus. Other viruses of unknown origin were also identified, including husavirus and human plasma-associated gemycircular virus (3, 4). Viral sequences of dietary origins were identified, including members of the plant-infecting *Virgaviridae* and *Betaflexiviridae* families and chicken anemia virus.

Also detected were reads with translated protein similarity to the Rep protein of circular Rep-encoding single-stranded DNA (CRESS-DNA) viral genomes (5), a large and diverse viral group that infects diverse eukaryote hosts, including vertebrates, fungi, plants, and insects (6–8), and is often found in environmental samples (8–10). A member of that clade (SsHADV) was shown to infect both a fungus and insects (11).

Here, the complete genome sequences of nine CRESS-DNA genomes were assembled using the contigs generated by the Ensemble program and the Geneious program version R10.1.2 (Auckland, New Zealand) to identify overlapping reads until direct repeats were generated at both extremities, which were then circularized into CRESS-DNA genomes.

Genomes were 2,548 to 2,853 bases long and encoded Rep and Cap proteins in opposite orientations. One genome was closely related to those of CRESS-DNA smacoviruses previously reported in fecal samples from diverse mammals (12, 13). The closest relatives were from captive chimpanzee (GenBank accession no. KT600069) and human (NC_026318) feces, with 95.4 to 96.2% Rep and 68.1 to 79.5% Cap identities. A similar tentative stem loop origin of replication was located 3′ of the *rep* gene.

The remaining genomes were closely related to each other, with Rep and Cap identities of 71.5 to 97.5% and 33.8 to 98.6%, respectively. The closest relatives in GenBank were CRESS-DNA virus from rhesus macaque feces (KU043400), dromedary stool-associated circular single-stranded DNA (ssDNA) virus (KM573765) (14), and bovine feces-associated circular DNA virus 1 (NC_030130) (15), with Rep protein identities ranging from 72.9 to 93.7%, 47.5 to 54.8%, and 44.8 to 49.5% and Cap protein identities ranging from 33.6 to 42.1%, 20.8 to 24.4%, and 22.5 to 27.4%, respectively. The intergenic regions 3′ of the *rep* gene all contained a stem loop with a potential origin consensus nanomer of NANNNTTAC. Rolling circle replication motifs I, II, and III and superfamily 3 helicase Walker A, B, and C motifs (7) were identified in all nine genomes. Based on gene orientations and location of the stem loop, all genomes correspond to type IV CRESS-DNA viruses (7).

The cellular host(s) or any pathogenic role for these feces-associated CRESS-DNA viruses remains unknown. Potential cell hosts include human enteric cells and ingested plant and/or animal food products.

### Accession number(s).

The complete genome sequences of hudisaviruses and human feces smacovirus 3 isolate P21 have been deposited at GenBank under the accession no. MF351513 through MF351520 and MF347417, respectively.
